# Structure, Activity and Function of the Protein Arginine Methyltransferase 6

**DOI:** 10.3390/life11090951

**Published:** 2021-09-11

**Authors:** Somlee Gupta, Rajashekar Varma Kadumuri, Anjali Kumari Singh, Sreenivas Chavali, Arunkumar Dhayalan

**Affiliations:** 1 Department of Biotechnology, Pondicherry University, Puducherry 605014, India; somleegupta@gmail.com; 2 Department of Biology, Indian Institute of Science Education and Research (IISER) Tirupati, Tirupati 517507, India; rajashekar@labs.iisertirupati.ac.in (R.V.K.); anjaliksingh@students.iisertirupati.ac.in (A.K.S.); schavali@iisertirupati.ac.in (S.C.)

**Keywords:** protein arginine methylation, PRMT6, post-translational modification, H3R2me2a, epigenetics, cancer

## Abstract

Members of the protein arginine methyltransferase (PRMT) family methylate the arginine residue(s) of several proteins and regulate a broad spectrum of cellular functions. Protein arginine methyltransferase 6 (PRMT6) is a type I PRMT that asymmetrically dimethylates the arginine residues of numerous substrate proteins. PRMT6 introduces asymmetric dimethylation modification in the histone 3 at arginine 2 (H3R2me2a) and facilitates epigenetic regulation of global gene expression. In addition to histones, PRMT6 methylates a wide range of cellular proteins and regulates their functions. Here, we discuss (i) the biochemical aspects of enzyme kinetics, (ii) the structural features of PRMT6 and (iii) the diverse functional outcomes of PRMT6 mediated arginine methylation. Finally, we highlight how dysregulation of PRMT6 is implicated in various types of cancers and response to viral infections.

## 1. Introduction

Protein arginine methyltransferases (PRMTs) are the family of enzymes which methylate the arginine residue(s) of substrate proteins and regulate a wide range of cellular events including transcription, splicing, translation, DNA damage responses and phase separation [1]. PRMTs are classified into three types depending on the nature of the methylarginine that they form. Type I PRMTs (PRMT1, PRMT2, PRMT3, PRMT4, PRMT6 and PRMT8) and Type II PRMTs (PRMT5 and PRMT9) generate asymmetric dimethylarginine and symmetric dimethylarginine, respectively, in addition to the monomethylarginine. Type III enzyme (PRMT7) exclusively generates monomethylarginines in proteins [2,3].

Protein arginine methyltransferase 6 (PRMT6) is a type I PRMT which is involved in epigenetic regulation of gene expression [4,5,6], alternative splicing [7,8], development and differentiation [9,10,11,12,13], DNA repair [14], cell proliferation and senescence [15,16,17,18,19,20], DNA methylation [21], mitosis [22,23], inflammation [24,25,26], innate antiviral immunity [27], spermatogenesis [28], transactivation of nuclear receptors [7,29,30] and cell signaling [31,32]. A high throughput yeast two-hybrid screening of PRMT6 identified 36 proteins as potential interaction partners of PRMT6 suggesting the role of PRMT6 in various additional, so far unidentified cellular functions [33]. In addition to these physiological functions, the dysregulation of PRMT6 is implicated in viral diseases [34,35,36,37,38,39,40,41], cancers [42] and cardiac hypertrophy [43].

The human PRMT6 gene, located on Chromosome 1, encodes for the 41.9 kDa PRMT6 enzyme. PRMT6 is predominantly localized to the nucleus, in stark contrast to PRMT3 and PRMT5 which are preponderantly cytosolic, while other PRMTs are found in both nucleus and cytosol [44]. PRMT6 is expressed in a wide range of tissues with high expression in kidney and testes [44]. The F-box proteins, FBXO24 and FBXW17, regulate the protein levels of PRMT6 by facilitating its proteasomal degradation [45,46]. PRMT6 generates asymmetric dimethylation modifications in histone 3 at arginine 2, arginine 17 and arginine 42 (H3R2me2a, H3R17me2a and H3R42me2a) [4,5,6,47,48] and in histone H2A at arginine 26 (H2AR26me2a) [49] and participates in the epigenetic regulation of gene expression. In addition to the histones, PRMT6 methylates a wide range of non-histone proteins and regulates various biological functions. In the following sections, we discuss the biochemical features, structural aspects, epigenetic functions of PRMT6, functional outcomes of PRMT6-mediated methylation of non- histone substrates and viral proteins and the role of PRMT6 in various types of cancers.

## 2. Biochemical Features

PRMT6 was identified as a protein arginine methyltransferase based on the presence of conserved catalytic core of PRMT family members. In vitro studies using the GST-GAR substrate, showed that the recombinant PRMT6 enzyme generates monomethylarginines and asymmetric dimethylarginines, establishing PRMT6 as a bona fide type I PRMT enzyme [44]. GST-GAR, an in vitro substrate used to investigate the enzymatic activity of PRMTs, is a GST fusion of N-terminal region of the human rRNA 2’-O-methyltransferase fibrillarin enzyme (amino acids 1 to 148). GST-GAR contains nine arginine residues in the sequence context of “RGG” and six arginine residues in the sequence context “RG” [50,51,52].

**Figure 1:**
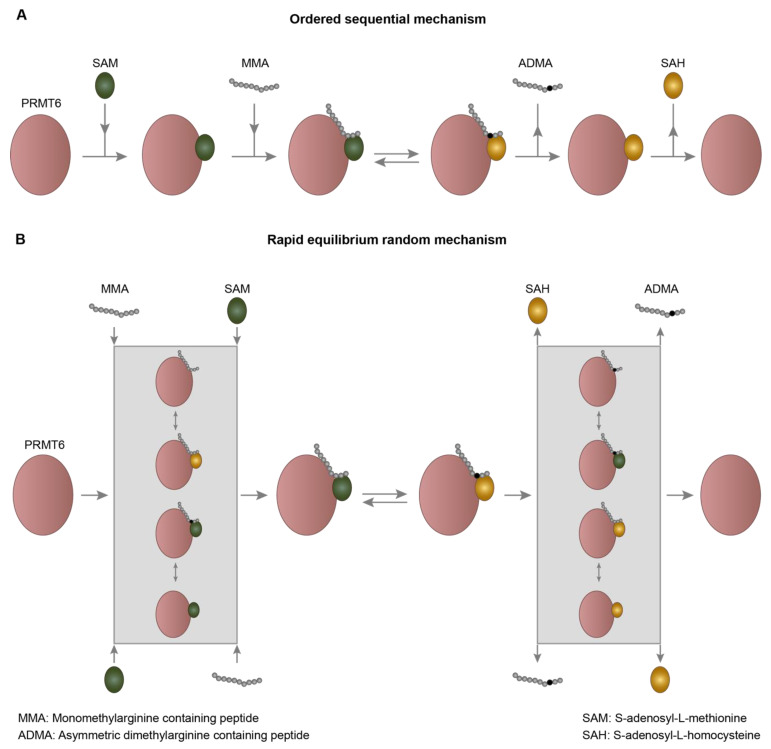
The proposed kinetic mechanisms of PRMT6 catalysis. Schematic representation of (**A**) Sequential ordered kinetic mechanism and (**B**) Rapid equilibrium random mechanism with dead-end complexes.

Kinetic studies of PRMT6 mediated methylation of GST-GAR substrate revealed that it generates asymmetric dimethylarginines in a processive manner [44]. Furthermore, investigation of kinetics of recombinant PRMT6 with substrate peptides containing a single arginine or monomethylarginine residue [53] revealed that PRMT6 follows an ordered sequential mechanism of catalysis in which the S-adenosyl-L-methionine (SAM) binds to the enzyme first, followed by the binding of the substrate peptide resulting in the formation of a ternary complex (Figure 1A) [53,54]. Consequent to the methyl group transfer, the methylated substrate peptide dissociates first from the enzyme followed by the S-adenosyl-L-homocysteine (SAH) [53,54]. The catalytic efficiency of PRMT6 on the monomethylarginine containing substrate peptides is much higher than that of the substrate peptides with unmethylated arginine [53]. Notably, peptide products with asymmetric dimethylarginine could not be detected in the enzymatic reaction of PRMT6 with unmethylated arginine containing peptides. Collectively these findings suggest that the PRMT6 enzyme generates asymmetric dimethylarginine in a distributive manner rather than introducing two methyl groups processively in a single arginine residue in a single binding event [53].

However, this ordered sequential kinetic mechanism of PRMT6 enzyme was contradicted by a subsequent study which showed that the pre-incubation of SAM with the PRMT6 enzyme did not alter the IC_50_ of C21 peptide on the activity of PRMT6 [55]. C21 is a modified H4 tail peptide which contains chloroacetamidine modified ornithine at arginine 3 position and this C21 peptide inhibits the enzymatic activity of PRMTs irreversibly with high selectivity for PRMT1 and modest selectivity for PRMT6 [55,56]. This suggests that the binding of the SAM with the enzyme is not a pre-requisite step for the substrate peptide binding, challenging the ordered sequential kinetic mechanism of PRMT6 [55]. This observation prompted further detailed investigations of the kinetic mechanism of PRMT6 catalysis. Product inhibition studies and usage of dead-end analogs revealed that PRMT6 follows a rapid equilibrium and random kinetic mechanism with the generation of dead-end complexes (Figure 1B) [55]. These studies led to the proposal that PRMT6 binds to the substrate peptides and SAM in a random fashion to produce a ternary complex and products dissociate randomly after the methyl group transfer to generate the free enzyme [55]. This necessitates detailed structural studies of PRMT6 in complex with the proper substrate peptide and thorough kinetic studies to resolve this conflict and delineate the actual kinetic mechanism of PRMT6 catalysis.

In addition to methylating its substrates, PRMT6 also undergoes automethylation at Arg35. This automethylation of PRMT6 regulates its stability and anti-HIV1 function without affecting its catalytic activity [44,57]. High throughput mass spectrometry analysis of PRMT6 activity on the H3 peptides carrying different amino acid exchanges at different positions showed that PRMT6 is relatively a non-specific enzyme in vitro as it tolerated most of the amino acid substitutions, with preference for positively charged amino acids and bulkier amino acids around the target arginine. PRMT6 exhibits a higher preference to methylate RG or RGK motif containing peptides more efficiently than the RGG motif containing peptides in vitro [58]. Contrarily, there is a higher preference of PRMT1 to methylate RGG motif containing substrates, while PRMT4 shows a preference to methylate the substrates in which the target arginine is flanked by the proline residues [58,59,60,61].

Given that the PRMT6 exhibits a relaxed substrate specificity, it is highly likely that it might methylate several hitherto unidentified substrate proteins, including the substrates of other PRMTs. Indeed, an in vitro methylation assay on rat cell extracts revealed that PRMT1, PRMT4 and PRMT6 methylate some common substrate proteins, alongside protein substrates that are unique to each of these PRMTs [44]. Both PRMT4 and PRMT6 are involved in the generation of asymmetric dimethylation at Arg17 of histone 3 (H3R17me2a) and asymmetric dimethylation at Arg42 of histone 3 (H3R42me2a) in vivo [47,48]. Examples of common substrates include (i) the type1A topoisomerase enzyme, TOP3B, which is methylated by PRMT1, PRMT3 and PRMT6 enzymes [62] and (ii) the cell cycle inhibitor, p16 is methylated PRMT1, PRMT4 and PRMT6 enzymes [15]. All these observations suggest that PRMT6 can also methylate the substrates of other PRMTs in addition to its unique substrates. Nevertheless, the biochemical and structural mechanisms underlying how overlapping substrates are methylated by different PRMTs remains elusive.

## 3. Structural Attributes

**Figure 2:**
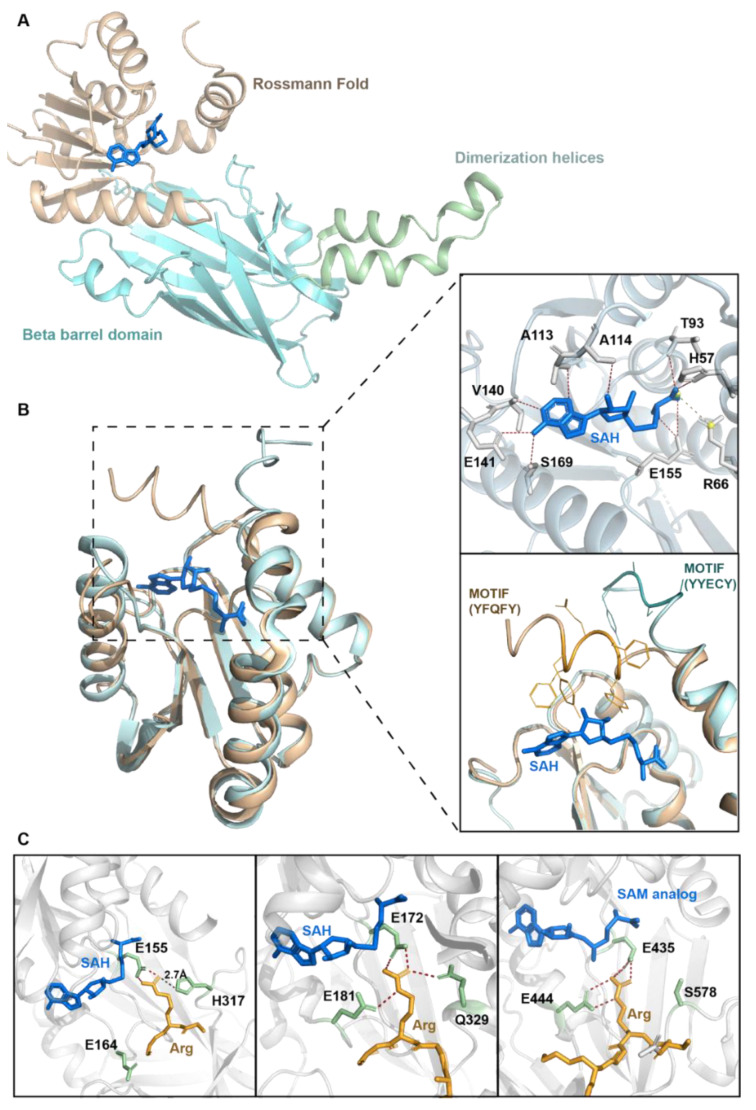
Structural attributes of PRMT6. (**A**) Crystal structure of the human PRMT6 (PDBID: 6W6D) in complex with SAH (marine blue). (**B**) Structural superimposition of the Rossmann fold region of PRMT6 (PDBID: 6W6D, cyan), PRMT4 (PDBID: 6IZQ, brown). Top inset shows the detailed interactions between PRMT6 and SAH (Top; PDBID: 6W6D). Polar contacts (red) and salt bridges (yellow) are displayed as dashed lines. PRMT6 is represented in cartoon and side chains of the residues (white) interacting with S-adenosyl-L-homocysteine (SAH; marine blue) are represented as sticks. Bottom inset shows the structural superimposition of the conserved motif from PRMT6 (Motif: YYECY) and PRMT4 (Motif: YFQFY). The sidechains of the motif residues are highlighted as wire-representations. (**C**) Structural comparison of the human PRMT6 (Left panel; PDBID: 5HZM) with *T. brucei* PRMT7 (Middle panel; PDBID: 4M38) and human PRMT5 (Right panel; PDBID: 4GQB) highlighting the active site in complex with the arginine (orange). SAH in the first two panels and S-adenosyl-L-methionine (SAM) analog in the third panel have been highlighted in marine blue. For the PRMT6 (left panel), arginine is modeled into the active site of human PRMT6 (PDBID: 5HZM) by superimposing the *T. brucei* PRMT7 structure (PDBID: 4M38). Distance between His317 and arginine Nη2 atom (left panel) is shown as black colored dashed line. Polar contacts are displayed as red colored dashed lines. Interacting residues side chains are represented as sticks (green).

PRMT6 of *Trypanosoma brucei*, mouse and human in complex with sinefungin (SNF) or SAH and/or short substrate peptide have been structurally characterized [63,64,65]. Mouse PRMT6 shares 91.5% sequence homology with human PRMT6 and 29% sequence homology with *T. brucei* PRMT6 [64]. All members of the PRMT family share a conserved catalytic PRMT core region with variable N-terminal regions. The overall structure of human PRMT6 is similar to that of other PRMT family members. PRMT6 consists of three structural components viz. (i) the N-terminal Rossmann fold, which contains the SAM binding pocket, (ii) the C-terminal β-barrel domain and (iii) a dimerization helix, which are located between the β6 strand and β7 strand of the C-terminal β-barrel domain (Figure 2A). A conserved proline (Pro186) in cis-conformation connects the N-terminal Rossmann fold and the C-terminal β-barrel domain [64,65]. The invariant residues in the SAM binding pocket of Rossmann fold interacts with the homocysteine carboxylate, adenine ring and ribose of SAH through hydrogen bonds and salt bridges [65] (Figure 2B).

Structural comparison of PRMT6 with other type I PRMT structures revealed two interesting features of PRMT6: (i) The conserved aromatic motif Y(F/Y)xxY at the N-terminal region covers the adenosine moiety of SAH as a lid in case of PRMT3 and PRMT4 structures [66,67,68], while in PRMT6 and PRMT1 this motif is positioned outwards from the SAH binding pocket [65,69,70] (Figure 2B). Since this aromatic motif is important for the SAH binding and catalysis of PRMT1 [70], it might adopt different conformations dynamically to facilitate the release of SAH during catalysis of PRMT1 and PRMT6. (ii) Similar to other type I PRMT members, PRMT6 also forms a dimer through the interaction of dimerization arm helices of one monomer with the N-terminal helices and the helices of Rossmann fold of the other monomer [65]. The dimerization arm of PRMT6 exhibits a different conformation and forms a flat ring dimer structure with a central cavity in contrast to the concave surface cavity formed by the other type I PRMT dimers [65,70,71]. This unique arrangement of the central cavity of PRMT6 dimer might influence the substrate selectivity of PRMT6.

Superimposition of the *T. brucei* PRMT7 (TbPRMT7) structure (PDBID: 4M38) [72] and modelling of the arginine residue into the active site of the human PRMT6 structure (PDBID: 5HZM) [65] revealed that the conserved glutamate Glu155 in the active site forms a hydrogen bond with the substrate arginine residue (Figure 2C). However, the other conserved glutamate, Glu164, which is part of the double E loop is directed outward from arginine in the active site. An outward direction of Glu164 was also observed upon the superimposition of the PRMT1-arginine-SAH complex structure [69] on the PRMT6 structure [65]. The corresponding glutamate Glu181 of TbPRMT7 and Glu444 of PRMT5 forms a hydrogen bond with the target arginine in TbPRMT7-H4R3 peptide-SAH and PRMT5-H4R3 peptide-SAH complexes respectively (Figure 2C) [72,73]. Moreover, the Glu164 residue points towards the active site in PRMT6 in complex with SAH and the inhibitor, EPZ0204111 [65]. Collectively, these observations suggest that the conserved Glu164 residue of the double E loop points towards the target arginine and forms a hydrogen bond or it points away from the active site, and this flexible nature of this residue might be important for the catalysis.

The THW motif, present in the loop that connects the β11 and β12 of the β-barrel domain, is located in the active site of PRMT6 and is conserved among the type I PRMTs. His317 of the THW motif is likely to form a hydrogen bond with the Nη2 atom of the target arginine as it is within the distance of 2.7 Å (Figure 2C). This might impede the swapping of Nη2 and methyl Nη1 positions and thus preclude symmetric dimethylation. The corresponding residue in TbPRMT7 complex, Gln329, also forms a hydrogen bond with the Nη2 atom of the target arginine in a similar manner and hence prevents the symmetric dimethylation (Figure 2C) [65,72]. However, in case of the type II PRMT5 enzyme complex, the corresponding residue of His317 is Ser578, which does not form a hydrogen bond with Nη2 atom of the target arginine as it points away from the target arginine (Figure 2C). Such an arrangement allows the swapping of Nη2 and methyl Nη1 positions and facilitates the generation of symmetric dimethyl arginine [65,73]. Taken together, these findings and comparisons provide the plausible structural basis for the mechanism underlying PRMT6 generation of asymmetric dimethyl arginine.

## 4. Biological Roles of PRMT6

### 4.1. Epigenetic Functions of PRMT6

Post-translational modifications of the histone tails regulate the chromatin structure and control various chromatin-dependent processes including gene expression. PRMT6 is the major enzyme that generates asymmetric dimethylation at Arg2 of histone 3 (H3R2me2a) in vivo and regulates the global levels of H3R2me2a [4,5,6]. PRMT6-mediated H3R2me2a modifications are enriched in the gene body and promoter regions of inactive genes and inversely correlate with active H3K4me3 (trimethylation of histone 3 at Lys4) modifications in the promoter regions. The H3R2me2a modification is a repressive mark as its presence in the promoters negatively correlates with the transcript levels of the associated genes [4]. Not surprisingly, presence of the active histone mark H3K4me3 inhibits the PRMT6 activity on the H3 peptides [4,5]. The SET1/MLL family of enzymes catalyze the formation of the active H3K4me3 modification and these enzymes function as a complex with other protein factors [74,75,76,77]. The MLL complex does not show activity on the H3 peptides with H3R2me2a modification [4,6]. These findings explain the observed counter-correlation between the H3K4me3 and H3R2me2a modifications and the corresponding roles of methyltransferases at the promoter regions.

The majority of the reader proteins which recognize the H3K4me3 modification exhibit a reduced or non-detectable binding with the H3 peptides carrying the dual H3K4me3 and H3R2me2a modifications. This indicates that the presence of H3R2me2a modification inhibits the recognition of H3K4me3 modification by the reader proteins [5]. MLL complex activates the expression of HoxA genes and Myc targets genes by generating H3K4me3 modifications at their promoters [76,78,79,80] and the PRMT6 down regulates the expression of these genes [6]. The over-expression of PRMT6 represses the expression of HoxA2 by increasing the level of H3R2me2 marks and reducing H3K4me3 modifications near the transcription start site of the HoxA2 gene [6] (Figure 2A).

**Figure 3:**
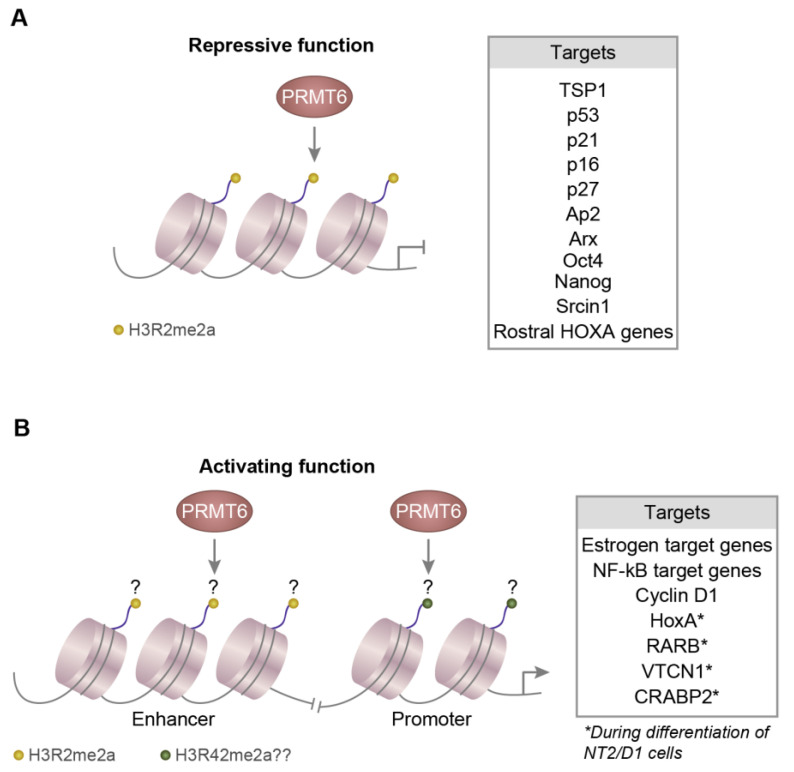
PRMT6 mediated epigenetic regulation of gene expression. (**A**) Schema highlighting the repressive role of PRMT6. PRMT6 generates H3R2me2a modifications at the promoters of the target genes and suppresses their expression. (**B**) Schematic representation of the transcriptional activation function of PRMT6. PRMT6 generates H3R2me2a modifications at the enhancers and probably H3R42me2a modifications at the promoters of the target genes and activates their expression. ‘?’ indicates that the highlighted histone modifications are putative and need further characterization.

PRMT6 mediated H3R2me2a modifications tend to co-occur with H3K27me3 in a subset of silent gene promoters [12,80,81]. PRMT6 interacts with the polycomb repressive complexes (PRC) and silences the transcription of rostral *HOXA* genes by generating H3R2me2a and H3K27me3 modifications in their promoters [82]. Thrombospondin-1 (TSP-1) is a secretory protein which inhibits angiogenesis strongly and negatively affects cell migration [83,84,85]. In the U2OS osteosarcoma cells, PRMT6 has been shown to down regulate the expression of TSP-1 by introducing H3R2me2a modifications and reducing the active H3K4me3 modification in the TSP-1 promoter regions (Figure 3A). This negative regulation of TSP-1 by PRMT6 increases the migration and invasive properties of the U2OS cells [85]. However, this effect appears to be cell/cancer-type specific, as the over-expression of PRMT6 in human estrogen-sensitive breast cancer cells (MCF7) and the human prostate cancer cells (PC3) increases the TSP-1 expression and hence inhibits the movement and invasion of cancer cells [86]. Additional studies are required to resolve this conundrum as to why enhanced levels of PRMT6 have opposing effects in different cancers.

From a molecular point of view, PRMT6 promotes cell proliferation and contributes to the tumorigenic properties and prevents premature cellular senescence by downregulating the expression of tumor suppressor genes, p53, p21, p16 and p27. PRMT6 downregulates these tumor suppressors by generating the repressive H3R2me2a modifications in their promoters and other regulatory regions [16,17,20,87]. PRMT6 regulates adipocyte differentiation by interacting with PPARγ and inhibiting its functions. PRMT6 suppresses the expression of the PPARγ target gene, adipocyte protein 2 (Ap2) by binding to the PPAR responsive regulatory element in the promoter of the Ap2 along with PPARγ and by adding repressive H3R2me2a modifications [88]. Aristaless Related Homeobox (Arx) is a lineage determining gene which is expressed exclusively in the pancreatic α cells. DNA methylation and PRMT6-mediated H3R2me2a modifications in the upstream regulatory regions of Arx gene suppresses its expression in pancreatic β cells [89]. Repeated cocaine exposure decreases the PRMT6 in dopamine D2 expressing medium spiny neurons (D2-MSNs) present in the nucleus accumbens of the basal forebrain. This leads to the decrease of H3R2me2a modifications and an increase in H3K4me3 modifications in the promoter region of Src kinase signaling inhibitor 1 (Srcin1) which in turn upregulates the Srcin 1 expression. The elevated levels of Srcin 1 inhibits the Src signaling, thereby reducing cocaine reward and the intent for the self-administration of cocaine [90] (Figure 3A).

PRMT6 and the associated H3R2me2a modifications increase globally during the differentiation of the mouse embryonic stem cells. Specifically, PRMT6 regulates the expression of the pluripotency genes, Oct4 and Nanog by modulating the levels of H3R2me2a and H3K4me3 modifications at their promoter regions [10]. During the megakaryocytic/erythroid lineage bifurcation of common hematopoietic progenitor cells, PRMT6 facilitates megakaryocytic differentiation by inhibiting the expression of erythroid genes. In the CD34 positive hematopoietic progenitor cells, the transcription factor RUNX1 interacts with PRMT6 and establishes the H3R2me2a modifications at the promoters of megakaryocytic genes [12,13]. During the megakaryocytic differentiation of the progenitor cells, PRMT6 dissociates from the RUNX1 co-repressor complex and facilitates the expression of megakaryocytic genes [12,13]. In addition, PRMT6 inhibits the expression of erythroid genes by generating the repressive H3R2me2a modifications in their promoter regions during megakaryocytic differentiation of the progenitor cells [91] (Figure 3A).

UHRF1 is the multi-domain protein factor which facilitates the recruitment of DNMT1 to the hemi-methylated CpG sites and the maintenance of DNA methylation pattern [92,93]. PRMT6 mediated H3R2me2a modifications inhibit the binding of UHRF1 to the chromatin which in turn negatively affects the DNA methylation by DNMT1 [21,94,95,96,97]. The high levels of PRMT6 in cancer cells lead to the global DNA hypomethylation and contribute to the carcinogenesis, possibly through the passive DNA demethylation [21]. The H3R2me2a modifications generated by PRMT6 play an important role in chromosome condensation during mitosis, because H3R2me2a recruits chromosomal passenger complex (CPC) to the chromosome upon mitotic entry and augments the H3S10 phosphorylation by Aurora B kinase which in turn facilitates the chromosome condensation [22].

In addition to the repressive epigenetic roles, PRMT6 also functions as a co-activator for steroid hormone receptors and this co-activator function requires the methyltransferase activity of PRMT6. PRMT6 promotes the expression of estrogen target genes and cell proliferation in an estrogen-dependent manner in MCF7 cells [7]. PRMT6 interacts with the transcription factor NF-κB and serves as co-activator to facilitate the expression of NF-κB target genes [24]. The transcription factor LEF1 activates the expression of cyclin D1 by recruiting PRMT6 to the promoter of cyclin D1 [98]. This co-activator function of PRMT6 might be due to the ability of PRMT6 to generate the active H3R42me2a modifications as well in the chromatin (Figure 3B).

A recent thorough genome wide study of PRMT6 mediated H3R2me2a modification in human embryonal carcinoma NT2/D1 cells strikingly revealed that this modification is associated with promoters, transcriptional start site and enhancer elements of active genes rather than repressed genes [99]. The promoter and TSS site associated H3R2me2a modifications suppress the transcription of the associated active genes by preventing the generation of active H3K4me3 modifications at these locations. However, the enhancer associated H3R2me2a modifications activate the transcription of the associated genes by facilitating the deposition of H3K4me1 and H3K27ac modifications [99]. Interestingly, the PRMT6 generated H3R2me2a modifications tend not to co-occur at the promoter and enhancer regions of the same genes [99]. Thus, the transcriptional outcome of H3R2me2a modifications is dependent on the genomic location of these modifications (Figure 3B).

In addition to H3R2 methylation, PRMT6 also methylates H4 at Arg3 residue (H4R3me2a) in vitro [6], H3 at Arg42 residue (H3R42me2a) [47] and H2A at Arg29 residue (H2AR29me2a) both in vitro and in vivo [49]. The PRMT6 mediated H2AR29me2a modifications are enriched in the promoter regions of the specific genes which are down regulated [49]. PRMT6 also methylates H3 at Arg17 residue (H3R17me2a) in vitro alongside the PRMT4 enzyme. H3R17me2a levels are increased during the mitosis [100] and this increase of H3R17me2a requires both PRMT4 and PRMT6 enzymes. Moreover, over-expression of PRMT6 increases the H3R17me2a modifications globally. All these findings suggest that PRMT6 is also involved in the deposition of H3R17me2a alongside PRMT4 [48]. Thus, PRMT6 mediated histone modifications affect diverse biological processes by regulating the gene expression, deposition of other histone modifications and DNA methylation.

### 4.2. Functional Outcomes of PRMT6 Mediated Methylation of Its Substrates

Below, we will discuss the different non-histone substrates of PRMT6, identified through targeted molecular and/or biochemical studies and their functional consequences, as applicable. Firstly, we will discuss the human substrates and then shed light on the current understanding of the viral proteins that are methylated by PRMT6 (Table 1).

#### 4.2.1. Substrates in Human Cells

(*i*). *DNA repair protein-DNA Polymerase β*

DNA Polymerase β plays an important role in the base excision repair [110,111,112]. PRMT6 interacts with and methylates DNA Polymerase β at Arg83 and Arg152 residues. Methylation of DNA Polymerase β by PRMT6 enhances its polymerase activity by increasing its processivity. The residues which are methylated by PRMT6 are important for efficient repair of DNA damage introduced by the alkylating agents [14].

(*ii*). *Chromatin modifiers-HMGA1a, SIRT7*

HMGA1 proteins are nuclear non-histone proteins which regulate the chromatin structure and gene expression [113,114,115]. PRMT6 has been shown to methylate HMGA1a protein at Arg57 and Arg59 residues, both in vitro and in vivo [101,102,103]. These PRMT6 target residues are located in the second AT hook region of HMGA1a which is important for the binding of HMGA1a with DNA, suggesting that PRMT6 mediated methylation of HMGA1a might affect its binding with the DNA [102,103].

SIRT7, a deacetylase, catalyzes the removal of the acetylation modification of histone 3 at Lys18 (H3K18ac) and regulates many cellular processes including mitochondrial biogenesis [116,117,118,119,120,121]. PRMT6 interacts with SIRT7 and methylates it at Arg388. This methylation inhibits the deacetylase activity of SIRT7 and positively regulates mitochondrial biogenesis. The glucose availability regulates the PRMT6-mediated methylation of SIRT7 in an AMPK dependent manner [106]. Thus, the methylation of SIRT7 by PRMT6 serves as a link that connects mitochondrial biogenesis with glucose levels [106].

(*iii*). *Transcription regulators-CRTC2, FOXO3, GPS2 and TOP3B*

The transcription factor CREB (cAMP response element binding protein) and the CREB-regulated transcriptional coactivator 2 (CRTC2) activate the expression of gluconeogenic enzymes in the liver during fasting [122,123,124,125]. PRMT6 interacts with CRTC2 and generates asymmetric dimethylation modifications at several arginine residues of CRTC2. These PRMT6-mediated methylations of CRTC2 aid its association with CREB at the promoter regions and enhance the expression of gluconeogenic enzymes in hepatocytes [104,126].

FOXO3 is a multifunctional transcription factor and is implicated in various biological processes including gluconeogenesis, DNA repair, cell cycle autophagy, redox balance and proteostasis [127,128]. It is also involved in muscle atrophy by inducing protein degradation through the expression of muscle specific ubiquitin ligases [129,130,131]. The muscle specific knock out of PRMT1 increases the expression of autophagic markers and muscle specific ubiquitin ligases and promotes the muscle atrophy. Depletion of PRMT1 upregulates the expression of PRMT6 in muscle cells which in turn methylates FOXO3 at Arg118, Arg218 and Arg249. The PRMT6 mediated methylation of FOXO3 enhances its activity and contributes to the muscle atrophy [107].

G protein pathway suppressor 2 (GPS2) is a transcriptional regulator and is implicated in several cellular processes including cell cycle, apoptosis, bile acid synthesis and inflammation [132,133,134,135,136,137,138]. Methylation of GPS2 at Arg312 and Arg323 residues by PRMT6 promotes the association of GPS2 with TBL1, which inhibits the proteasomal degradation of GPS2, thereby leading to its enhanced stability, with implications across diverse cellular functions [105].

TOP3B is a type1A topoisomerase enzyme which resolves the topological strains of both DNA and RNA [139,140]. The histone arginine methylation reader protein, TDRD3 interacts with TOP3B and recruits it to the target chromatin regions wherein TOP3B executes its functions [141,142]. PRMT1, PRMT3 and PRMT6 methylates the Arg833 and Arg835 in the C-terminal region of TOP3B. The methylation of TOP3B by PRMT6 is required for (i) the efficient relaxation of supercoiled DNA and preventing the R-loop formation during transcription and (ii) localization of TOP3B to the stress granules [62]. The Tudor domain of TDRD3 recognizes the methylarginines of TOP3B which enhances TOP3B-TDRD3 interaction and facilitates the localization of TOP3B to stress granules [62].

(*iv*). *Cell cycle inhibitors and tumor suppressor-P16, P21 and PTEN*

In addition to suppressing the expression of the cell cycle inhibitors p16 and p21 through epigenetic modifications [16,17], PRMT6 also methylates p16 and p21 and negatively regulates their functions. PRMT6 methylates p16 at Arg22, Arg131 and Arg138 residues and promotes the cell cycle by inhibiting the interaction of p16 with CDK4 [15,18]. PRMT6 has been shown to methylate p21 at Arg156 residue both in vitro and in vivo. This methylation promotes the phosphorylation of p21 resulting in the accumulation of the protein in cytoplasm [19].

The phosphatase PTEN is a tumor suppressor gene which is often mutated in cancers [143,144]. PTEN negatively regulates AKT signaling by dephosphorylating the phosphatidylinositol-3,4,5-trisphosphate (PIP3) [145,146]. PRMT6 methylates PTEN at Arg135, which is frequently mutated in cancer. The methylation of PTEN by PRMT6 is required for the efficient suppression of AKT signaling by PTEN and modulates the global alternative splicing of pre-mRNAs [31].

(*v*). *Hormonal receptors-ERα and AR*

PRMT6 interacts and methylates the nuclear receptor ERα. PRMT6 promotes the estrogen-dependent and estrogen-independent activities of ERα in a methyltransferase activity dependent and independent manner, respectively. The interaction of PRMT6 with ERα inhibits the ERα-HSP90 interaction and promotes ligand independent functions of ERα [30]. Though the methyltransferase activity of PRMT6 is required for the enhancement of estrogen dependent activities of ERα [7,30], the precise functional outcome(s) of the PRMT6 mediated methylation of ERα is unknown.

The expansion of a polyglutamine tract in the androgen receptor (AR) causes the spinobulbar muscular atrophy (SBMA) disease [147,148,149,150]. PRMT6 promotes the hormone dependent transactivation function of normal AR as well as the polyglutamine expanded AR (mutant AR) in a methyltransferase activity dependent manner. The enhancement of transactivation function by PRMT6 is more pronounced for mutant AR compared to the normal AR [29]. PRMT6 forms a complex with AR and methylates AR at the arginine residues in the Akt consensus motif and inhibits the phosphorylation of AR by the Akt. PRMT6 contributes to the toxicity of polyglutamine expanded AR through its enhanced transactivation and interaction with the mutant AR [29].

(*vi*). *Scaffold protein-HTT*

The polyglutamine expansion in the huntingtin (HTT) protein causes the neurodegenerative disorder Huntington’s disease (HD). The scaffold protein, HTT facilitates the transport of organelles in the axons and dendrites of the neurons [108,151]. The polyglutamine expansion in the mutant HTT affects its axonal transport [152,153,154,155]. PRMT6 interacts with HTT protein and deposits asymmetric dimethylation at Arg118. PRMT6 mediated methylation of HTT is required for the efficient axonal transport of vesicles and the viability of neurons [108]. Overexpression of PRMT6 in HD cells rescued the axonal trafficking and the neuronal viability [108].

Besides the aforementioned substrates, PRMT6 also methylates snRNPB, MIF, TUBB2A and HSJ-2 in vitro_._ However, the functional consequences of these methylations are unknown and remain to be investigated [33].

#### 4.2.2. Viral Substrates

Several proteins of human immunodeficiency virus type I (HIV-1) are methylated by PRMT6, as part of the host response to suppress viral infection/propagation. For instance, PRMT6 interacts with the Tat protein of HIV-1, a transcriptional activator that stimulates the transcription of the viral genes and facilitates the viral replication [156], and methylates it at Arg52 and Arg53 positions [34,35,36]. This PRMT6 mediated methylation of Tat (i) increases its stability, (ii) excludes the Tat from the nucleolus and (iii) decreases its transactivation function resulting in the reduced production of viral particles [34,35,37,38]. Mechanistically, PRMT6 mediated methylation of Tat inhibits its interaction with Tat transactivation region (TAR) of HIV-1 RNA and inhibits formation of Tat-TAR-cyclin T1 ternary complex, resulting in the compromised transactivation function of Tat [35]. However, ectopic expression of PRMT6 in A549 cells and HeLa cells did not show inhibition of transactivation function of Tat [36] (Table 1).

Besides Tat protein, PRMT6 also methylates other HIV-1 viral proteins [39,40], including the Rev protein at its N-terminal arginine rich motif [39]. The Rev protein of HIV1 facilitates the nuclear export of intron containing viral RNAs [157,158,159]. PRMT6-mediated methylation of Rev decreases its binding with the Rev response element (RRE) of the viral RNA and inhibits the nuclear export function of the Rev [39]. The nucleocapsid protein (NC) of HIV-1 is methylated at Arg10 and Arg32 positions by PRMT6. The NC protein plays an important role in the packing of the HIV1 RNAs and in the annealing of tRNA Lys to the primer binding site of viral RNA [160,161,162,163,164,165,166]. PRMT6-mediated methylation of NC protein hampers its ability to anneal the tRNA Lys to the primer site of viral RNA [40] (Table 1).

The pUL69 protein of the human cytomegalovirus facilitates the export of the unspliced mRNAs from nucleus to the cytoplasm by interacting with host mRNA export factor UAP56 or URH49 [167,168]. PRMT6 interacts with pUL69 at its N-terminal region which is important for the pUL69-UAP56 or URH49 interactions [41]. It is possible that the pUL69-PRMT6 interaction might affect the mRNA export function of pUL69. PRMT6 also methylates the N-terminal region of pUL69 [41] but the functional consequence of this methylation is hitherto not known (Table 1).

Taken together, these findings demonstrate that PRMT6 restricts HIV1 replication and propagation of viral particles by methylating the viral proteins and inhibiting their functions. Hence, any intervention strategy which maintains the optimal level of PRMT6 in cells such as preventing the proteasomal degradation of PRMT6 or promoting the expression of PRMT6 might serve well to restrict HIV1 infection and hence disease progression.

## 5. Role of PRMT6 in Cancers

**Figure 4:**
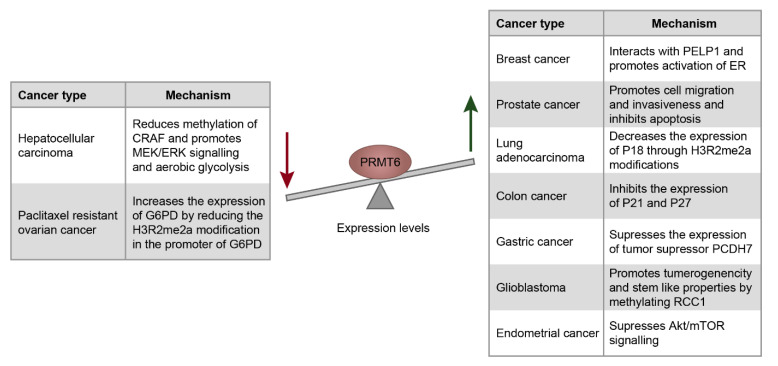
The role of PRMT6 in cancers. Schema representing the role of PRMT6 dysregulation in different cancers. The green arrow represents the upregulation of PRMT6 levels, while red arrow indicates the down regulation of PRMT6 levels in the corresponding cancer types, provided alongside in the table.

PRMT6 levels are elevated in several types of cancers and the depletion of PRMT6 inhibits the proliferation of lung and bladder cancer cells [42]. In the following sections, we will discuss the role of PRMT6 in different cancer types (Figure 4).

### 5.1. Breast, Prostate, Endometrial and Ovarian Cancers

PRMT6-mediated gene expression and alternative splicing changes are implicated in the pathophysiology of breast cancer [8]. The protooncogene PELP1 interacts with PRMT6 and promotes the activation of estrogen receptor, cell proliferation and clonogenic capacity of the breast cancer cells [169]. Overexpression of PRMT6 in the mammary glands of the mouse models promoted the tumorigenesis of mammary glands and deregulated Akt signaling in mammary epithelial cells [170].

PRMT6 levels are up-regulated in prostate cancer [171,172] and in endometrial cancer [173]. Depletion of PRMT6 in prostate cancer cells promotes apoptosis and decreases the cell migration and invasiveness properties [172]. Depletion of PRMT6 inhibits the endometrial cancer cell proliferation and migration by negatively regulating AKT/mTOR signaling [173].

Glucose-6-phosphate dehydrogenase (G6PD) levels are higher in paclitaxel resistant ovarian cancer cells compared to that of paclitaxel sensitive cancer cells. Depletion of G6PD in the paclitaxel resistant ovarian cancer cells increases its sensitivity to the paclitaxel treatment. PRMT6 is less abundant in paclitaxel resistant ovarian cancer cells compared to the sensitive cells. These low levels of PRMT6 lead to reduction of the repressive H3R2me2a modifications in the promoter region of G6PD resulting in the enhanced expression of G6PD which contributes to the paclitaxel resistance of the ovarian cancer cells [174].

### 5.2. Lung Cancer

PRMT6 levels are elevated in lung cancer [42,175]. Over-expression of PRMT6 in the lungs of mouse model enhanced the cell proliferation, promoting the lung tumor growth upon induction with a chemical carcinogen. PRMT6 interacts with interleukin enhancer binding protein 2 (ILF2) and activates the tumor associated macrophages [175]. PRMT6 levels are higher in the lung tissues of the patients with lung adenocarcinoma, which is associated with poor clinical outcomes [176]. Increased levels of PRMT6 reduces the expression of the cell cycle regulator p18 by increasing the amount of H3R2me2a and reducing the levels of H3K4me3 modifications. Depletion of PRMT6 activates the expression of p18 and inhibits the proliferation of lung adenocarcinoma cells [176].

### 5.3. Colon and Gastric Cancers

PRMT6 levels are elevated in colon cancer possibly due to the hypomethylation of PRMT6 promoter regions [177,178]. An abundance of PRMT6 is correlated with the shorter disease-free survival of colon cancer patients [178]. PPARα regulates the expression of DNMT1 and PRMT6 in the intestinal cells and protects against colon cancer. Loss or depletion of PPARα increases the levels of DNMT1 and PRMT6 which suppresses the expression of the cell cycle inhibitors p21 and p27 respectively and contributes to the colon carcinogenesis [179].

PRMT6 and H3R2me2a levels are elevated in gastric cancer (GC) and are correlated with poor prognosis. PRMT6 promotes the tumorigenicity and invasiveness of GC cells by silencing the expression of the tumor suppressor PCDH7 by depositing the repressive H3R2me2a marks at its promoter regions [180].

### 5.4. Glioblastoma

Regulator of chromosome condensin1 (RCC1) is a guanine nucleotide exchange factor for RAN-GTPase [181], which plays an important role in mitosis [182]. PRMT6 promotes the tumorigenicity and stem-like properties of the Glioblastoma stem cells (GSCs) by interacting with RCC1 and methylating it at Arg214. PRMT6-mediated methylation of RCC1 facilitates its association with chromatin and promotes mitosis through the generation of RAN-GTP [23,183].

### 5.5. Hepatocellular Carcinoma

RAF kinases activate the MEK/ERK signaling pathway and its dysregulation is implicated in cancers [184]. PRMT6 is downregulated in Hepatocellular carcinoma (HCC) and this downregulation is correlated with aggressive features of HCC. PRMT6 negatively regulates tumorigenic and stem-like properties of HCC. PRMT6 interacts and methylates CRAF at Arg100 which negatively regulates MEK/ERK signaling by inhibiting its interaction with RAS [32]. This negative regulation of MEK/ERK signaling also inhibits the aerobic glycolysis by preventing the ERK dependent nuclear localization of pyruvate kinase M2 (PKM2) [185]. In addition, the downregulation of PRMT6 promotes the autophagy and contributes to the tumorigenicity and cell survival in the tumor microenvironment. PRMT6 interacts with and methylates the co-chaperone Bcl-2, which is associated with athanogene 5 (BAG5) [186] at Arg15 and Arg24 and inhibits autophagy [109]. BAG5 forms a complex with the autophagic inducer, HSC70 [187,188] and the PRMT6-mediated methylation of BAG5 decreases the stability of HSC70 which in turn negatively regulates autophagy [109]. Thus, the downregulation of PRMT6 contributes to the tumorigenic and stem-like properties of HCC.

Taken together, the elevated levels of PRMT6 are linked to carcinogenesis of many different types of cancers mediated through methylation of histone or non-histone substrates and/or protein interactions. Not surprisingly, downregulation of PRMT6 across these cancer types leads to a reduction in cell proliferation and invasiveness through different mechanisms (Figure 4). Depletion or knock-out of PRMT6 reduced (i) the cell proliferation and clonogenic capacity of the breast cancer cells [169], (ii) the cell migration and invasiveness of prostate cancer cells [172], (iii) endometrial cancer cell proliferation and migration [173], (iv) proliferation of lung adenocarcinoma cells [176] and (v) tumorigenic properties of gastric cancer cells [180]. It was reported that the inhibition of PRMT6 activity by the PRMT6 specific inhibitor EPZ020411 reduces the tumorigenicity of glioblastoma and improves its response to radiotherapy [23]. All these findings establish that PRMT6 is an important potential therapeutic target for various cancers. This necessitates studies that investigate the efficacy of PRMT6 specific inhibitors for cancer therapy. Contrary to these observations, downregulation of PRMT6 was observed in melanoma [189] and HCC [32].

## 6. Future Perspectives

PRMT6 downregulates the expression of the target genes by generating H3R2me2a modifications at their promoters. In addition to the repressive functions, PRMT6 also activates the expression of certain target genes. While the repressive functions of PRMT6 are well studied, we are beginning to understand the transcriptional activation functions of PRMT6. A combination of biochemical, structural and molecular studies are required to obtain a thorough understanding of the spatio-temporal context of the transcriptional activation functions of PRMT6. In addition to H3R2me2a modifications, PRMT6 also generates H2AR29me2a and H3R42me2a in vivo. We envision that future studies will delineate (i) the biological functions of these modifications, (ii) their regulatory roles on various cellular processes and (iii) their connections to the diseases. Since PRMT6 exhibits a relaxed substrate specificity in high throughput studies with peptides, it is very likely that PRMT6 has many more cellular protein substrates. This necessitates extensive efforts to identify the hitherto unidentified substrate proteins of PRMT6 and characterize the functional consequences of their PRMT6 mediated methylation. Structural studies of PRMT6 in complex with the substrate peptides and thorough kinetic studies are needed to delineate the actual kinetic mechanism of PRMT6 catalysis. The fact that PRMT6 methylates proteins of the RNA virus HIV-1 and modulates their functions necessitates a systematic investigation on the regulatory role(s) of PRMT6 on other RNA viral infections, especially SARS-CoV2. PRMT6 is elevated in several types of cancers and contributes to the tumorigenesis through various mechanisms. Hence, investigations based on the existing as well as novel potent and cell active PRMT6 inhibitors [190,191,192,193,194] for their therapeutic activities against various cancers especially in combination with the standard anti-cancer drugs could pave a way for targeted cancer interventions.

## Figures and Tables

**Table 1:** Non-histone substrate proteins of PRMT6 and the functional outcomes of their methylation by PRMT6.

S. No.	Substrate Proteins	Functional Outcome(s) of PRMT6 Mediated Methylation	Reference (PMIDs)
1.	HMGA1a	Might regulate the binding of HMGA1a with DNA [101,102,103].	16157300, 16293633, 17550233
2.	DNA Polymerase β	Increases the polymerase activity and facilitates the base excision repair [14].	16600869
3.	P16	Promotes the cell cycle by inhibiting P16 interaction with CDK4 [15,18].	23032699, 26622834
4.	P21	Promotes the phosphorylation of P21 and accumulation of P21 in cytoplasm [19].	26436589
5.	CRTC2	Promotes the CRTC2-CREG interaction and enhances the expression of gluconeogenic enzymes in hepatocytes [104].	24570487
6.	ERα	Might promote estrogen dependent functions of ERα [7,30].	24742914, 20047962
7.	AR	Inhibits the phosphorylation of AR and promotes the hormone dependent transactivation of AR [29].	25569348
8.	GPS2	Enhances the protein stability of GPS2 [105].	26070566
9.	TOP3B	Enhances the topoisomerase activity and facilitates TOP3B localization in stress granules [62].	29471495
10.	SIRT7	Inhibits SIRT7 deacetylase activity, thereby promoting mitochondrial biogenesis [106].	30420520
11.	FOXO3	Enhances FOXO3 activity and contributes to the muscle atrophy [107].	30653406
12.	PTEN	Inhibits Akt signaling and modulates global alternative splicing [31].	30886105
13.	HTT	Facilitates the axonal transport of organelles by HTT and enhances the neuronal viability [108].	33852844
14.	CRAF	Inhibits CRAF-RAS interaction and suppresses the MEK/ERK signaling in Hepatocellular Carcinoma (HCC) [32].	30332648
15.	BAG5	Promotes the degradation of the BAG5 interaction partner, HSC70 which in turn inhibits autophagy in HCC [109].	33186656
16.	RCC1	Facilitates association of RCC1 with chromatin and promotes mitosis in Glioblastoma [23].	33539787
17.	HIV1-TAT	(i) Increases TAT1 stability, (ii) excludes the Tat from the nucleolus and (iii) decreases its transactivation function [34,35,37,38].	19726520, 15596808, 26611710, 17267505
18.	HIV1-REV	Inhibits nuclear export function of REV [39].	17176473
19.	HIV1-Nucleocapsid protein (NC)	Decreases the capacity of NC to anneal the tRNA Lys to the primer site of viral RNA [40].	17415034
20.	pUL69 of human cytomegalovirus	The functional consequence of this methylation is unknown [41].	26178996

## Data Availability

Not applicable.
